# Oral Tongue Reconstruction with a Bozola Flap According to the Ansarin Glossectomies Classification

**DOI:** 10.3390/jcm14061965

**Published:** 2025-03-14

**Authors:** Giovanni Salzano, Francesco Ferragina, Stefan Cocis, Fabio Maglitto, Alfonso Manfuso, Chiara Copelli

**Affiliations:** 1 Maxillofacial Surgery Unit, Department of Neurosciences, Odontostomatological and Reproductive Sciences, Federico II University of Naples, 80131 Naples, Italy; giovannisalzanomd@gmail.com; 2 Maxillofacial Surgery Unit, Department of Experimental and Clinical Medicine, Renato Dulbecco Hospital, Magna Graecia University of Catanzaro, 88100 Catanzaro, Italy; 3 Maxillofacial Surgery Unit, Interdisciplinary Department of Medicine, Aldo Moro University of Bari, 70125 Bari, Italy; stefandr.cocis@gmail.com (S.C.); fabio.maglitto@policlinico.ba.it (F.M.); manfuso@inwind.it (A.M.); chiara.copelli@uniba.it (C.C.)

**Keywords:** maxillofacial surgery, oral cancer, tongue cancer, oral tongue reconstruction, reconstruction surgery, regional flap

## Abstract

**Background:** Myomucosal cheek flaps are currently considered the main reconstructive option for small to moderate oral cavity defects. Many reconstructive techniques following the resectioning of oral tongue squamous cell carcinoma (OTSCC) have been proposed over the years. **Methods:** We report a case of OTSCC treated surgically and reconstructed with Bozola flap, analyzing the advantages and disadvantages of this surgical technique. The defect was classified according to the glossectomy classification proposed by Ansarin. **Results:** We believe that the Bozola buccinator myomucosal flap is a viable alternative to free flaps for the reconstruction of certain oral tongue defects. **Conclusions:** Based on our experience, the Bozola flap is an appropriate primary option for T1–T2 and certain T3 OTSCC defects (excluding the tip) for I–II and IIIa glossectomies, as recorded in the Ansarin classification.

## 1. Introduction

The reconstruction of defects resulting from tongue tumor resection still represents a challenge for head and neck surgeons. Given the primary function of the tongue and its complex anatomy and physiology, the main objective, once appropriate oncologic resection has been accomplished, must be an aesthetic–functional restoration. Several surgical techniques have been suggested, including primary closure, second intention healing, and the use of local or free flaps based on the size of the defect [1,2,3,4]. Free flaps provide a large amount of viable tissue but may compromise the functional outcome. Often the transplanted tissues are too bulky and may not provide the same mobility, sensation, mobility, or consistency as the original one. It is preferable to apply local flaps that reconstruct like-to-like tissue whenever possible. Myomucosal cheek flaps are widely used, reliable, and possess extensive vascularization. The main feature of these flaps is to maintain mucous secretion and sensitivity, facilitating the rehabilitation of oral function in speaking and ingestion [5,6]. FAMM, Zhao, and Bozola flaps are distinguished by their vascular supply on the buccal or facial artery [5]. The buccinator myomucosal flap, also called the Bozola flap, is an axial flap with a buccal artery-based posterior pedicle [7,8].

The purpose of this study is to describe the use of the Bozola flap after the resection of an oral tongue squamous cell carcinoma (OTSCC). We will endeavor to provide specific guidelines on the situations in which you can perform this flap in accordance with the 2018 Ansarin glossectomies classification [9]. This reconstruction technique is notable for its simplicity and reliability. It is considered suitable for defects due to T1–T2 and some T3 of OTSCC. Complications arising from the technique are rare and the functional result is of a high quality. This is due to the fact that the technique avoids the need for a prosthesis for the shutter and the formation of any skin scar. It is therefore recommended that this technique be considered for use in the reconstruction of the tongue after oncological surgery.

## 2. Materials and Methods

### 2.1. The Ansarin Classification of Glossectomies

**Figure 1:**
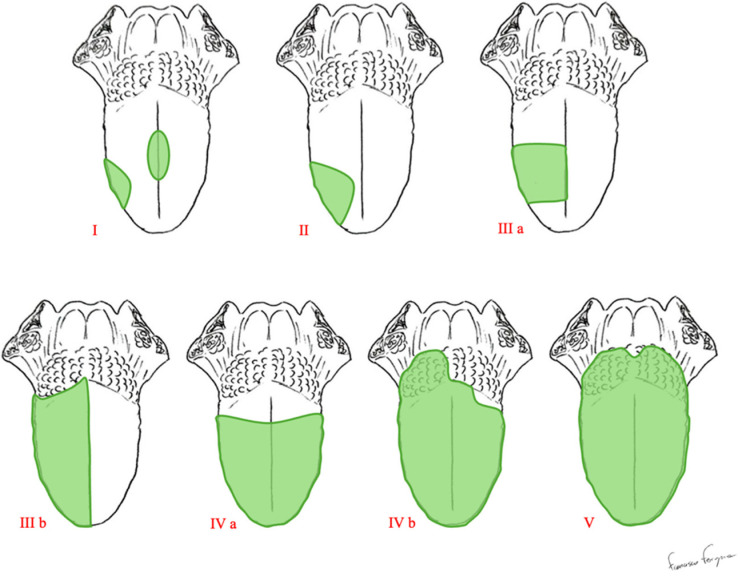
Classification of glossectomies proposed by Ansarin.

Ansarin proposed a classification of glossectomies into 5 groups based on the anatomical and functional components of the tongue [9]. The removal of these components depends on the extent of the tumor. Table 1 and Figure 1 show how glossectomy is classified according to Ansarin.

Type I involves the removal of the mucosa and submucosa, including a thin layer of the intrinsic muscles. Type II consists of removing the mucosa, submucosa, and intrinsic muscles up to the surface of the extrinsic muscles, with appropriate safe margins. Type IIIa extends to all of the ipsilateral extrinsic muscles, including the lingual artery and the lingual and hypoglossal nerves. The base of the ipsilateral tongue is preserved. Instead, the tip of the tongue can be preserved or not. Type IIIb includes the genioglossus muscle, hyoglossus muscle, styloglossus muscle, and the lower portion of the palatoglossus muscle. Medially, the midline raphe is included in the resection. The lingual nerve is resected as much as possible cranially, the hypoglossal nerve is removed after the ansa, and the lingual artery and vein are ligated in proximity to the hyoid bone. Type IVa is an anterior-subtotal glossectomy with the preservation of both sides of the tongue base; Type IVb extends to the ipsilateral base of the tongue. Type V is a total glossectomy; it includes the mobile tongue, both sides of the tongue base (transected at the level of the vallecula), intrinsic and extrinsic muscles, both lingual arteries, hypoglossal, lingual nerves, and the floor of the mouth.

### 2.2. The Anatomy of the Cheek

The cheek represents the lateral wall of the mouth. It consists of several anatomical structures: the mucosa, submucosa, buccinator muscle, masseter muscle, medial pterygoid muscle, buccal fat pad, and buccopharyngeal fascia. The complexity of its anatomy is indicative of the variety of its functions. It plays a crucial role not only in the aesthetic appearance of the face but also in chewing, phonation, and facial expression. The upper limit is at the level of the maxilla and the lower limit is at the level of the jaw. In addition, the anterior fibers of the buccinator muscle intertwine with some of the fibers of the orbicularis oris muscle. At the back, the cheek is inserted into the pterygomandibular raphe. The inner surface of the cheek is lined with thick and resistant mucosa, which is vital for withstanding the friction caused by mastication. This mucosa also encloses minor salivary glands. The parotid duct (Stensen’s duct) pierces the upper vestibular mucosa in front of the second upper molar. From the lower part of the parotid gland, the Stensen’s duct runs forward to cross the buccinator muscle. The buccinator muscle is categorized as a mimic muscle; however, its position, structure, and function are crucial for the processes of mastication and phonation. It originates from the alveolar process of the maxilla (at the upper molars), the pterygomandibular raphe, and the alveolar process of the jaw (at the lower molars). These fibers converge in front to fit into the labial commissure; here, they merge with the fibers of the orbicularis oris muscle and other mimic muscles, contributing to the functionality of the lips.

There are three main sources of arterial irrigation for the buccinator. The facial artery branches off in the front part of the muscle. The buccal artery branches off in the back part of the muscle. In addition, the posterosuperior alveolar artery gives a small branch to the buccinator. There are multiple and continuous anastomoses between all of these arteries.

The facial and buccal arteries both originate from the external carotid artery and are responsible for supplying different structures of the face, thereby contributing to the nourishment of muscles, skin, and oral mucosa.

The facial artery originates from the anterior portion of the external carotid artery, usually just above the lingual artery. The point of origin may vary slightly from individual to individual but is usually located at the lower edge of the jaw. After its origin, the facial artery follows a sinuous path that facilitates its adaptation to the movements of the face and jaw.

The buccal artery originates from the pterygoid portion of the maxillary artery. This point of origin is located in the proximity of the infratemporal trench. After its origin, the buccal artery moves forward and slightly downwards, passing through the buccinator muscle that protects it. This route makes it less exposed to direct trauma than other superficial arteries of the face.

The venous drainage is based on several veins originating from the lateral part of the muscle, all of which are tributaries to either a posterior main collector (the pterygoid plexus and the internal maxillary vein) or an anterior collector (the facial vein).

Muscle motor innervation comes from the facial nerve, specifically its temporal and cervical divisions, which converge near the buccal fat pad. The sensory innervation of both the muscle and buccal mucosa is due to the arborization of the inferior maxillary nerve.

### 2.3. Flap Harvesting

**Figure 2:**
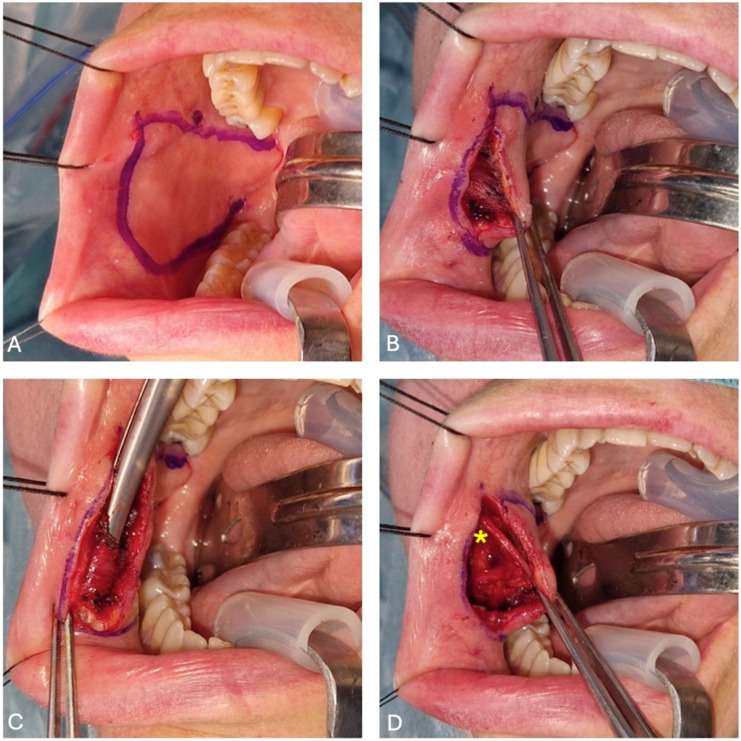
(**A**) Flap design and identification of Stensen’s duct papillae; (**B**) buccal mucosa dissection; (**C**) buccinator muscle identification through the scissors; and (**D**) facial artery (highlighted with the yellow asterisk) identification and cauterization.

The surgical procedure is performed under general anesthesia with nasal intubation. The flap is harvested from the same side as the defect. Certain steps are crucial, such as the identification of Stensen’s duct papillae and the vascular pedicle (the buccal artery that emerges from the internal maxillary artery, which ensures flap vascularization) by Doppler and the subsequent drawing of the flap over its trajectory. We draw the flap on the cheek mucosa (Figure 2A) in accordance with some specific landmarks: the upper margin is at least 0.5 cm below the Stensen’s duct papillae, to avoid the risk of an obstructive sialoadenitis; the anterior margin is at least 1 cm behind the oral commissure (exceeding this limit leads to an anesthetic scar retraction of the commissure); the posterior margin depends on the vascular pedicle and is made up by the pterygomandibular raphe where the vascular pedicle enters the flap; the lower margin depends on the size of the defect (the maximum size is 4–5 cm in the cephalon-caudal plane and 7 cm in the anteroposterior plane). At this stage, the flap margins are infiltrated with a solution of local anesthetic and 1% adrenaline; to facilitate tissue retraction, silk 2/0 sutures are applied to the lip commissure and the lateral portion of the upper and lower lips. We engrave, in the retro-commissural region, the distal portion of the flap (Figure 2B). The mucosa and the buccinator are cut and raised from the buccopharyngeal fascia in the anteroposterior plane (Figure 2C). The facial artery and its branches are identified and clipped (Figure 2D). It may be necessary to bind the anterior venous tributaries from the pterygoid plexus.

**Figure 3:**
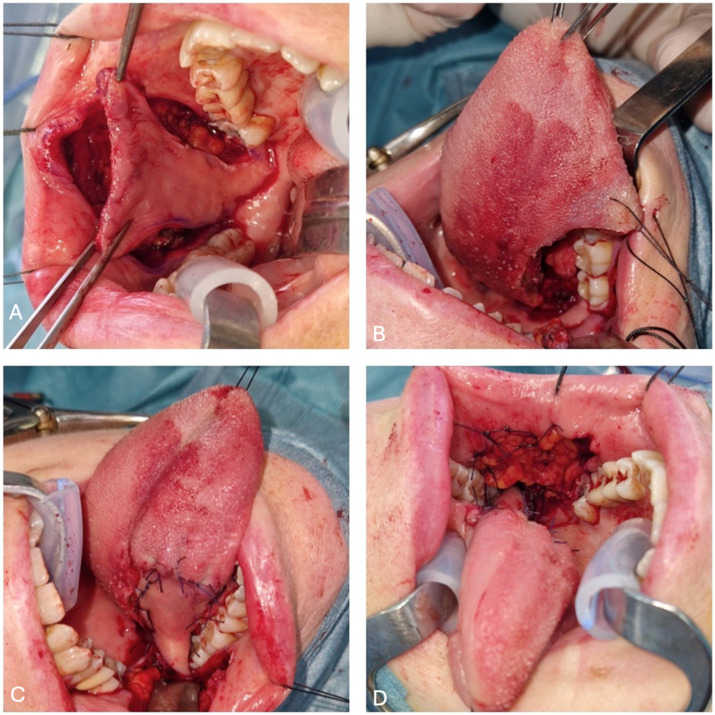
(**A**) Bozola flap placement from front to back; (**B**) defect size after IIIa right glossectomy; (**C**) flap pedicle and its arc of rotation; and (**D**) donor site reconstructed with fat pad.

The dissection proceeds posteriorly up to the pterygomandibular raphe (Figure 3A), following the buccal artery. The buccal artery, accompanying vein, and buccal nerve arise laterally at the posteroinferior aspect of the buccinator muscle. The flap, which is composed of mucosa, submucosa, and buccinator muscle, is trimmed based on the defect (Figure 3B). The oro-pharyngeal fascia must be preserved to prevent the herniation of the buccal fat pad and avoid injury to branches of the facial nerve.

The flap’s arc of rotation passes into the retromolar area of the jaw (Figure 3C) at the base of the vascular pedicle. During the rotation and transfer to the defect, great care should be taken to avoid forming excessive folds and twists in the pedicle. If there is tension on the flap, it will be necessary to increase the dissection of the pedicle at the base. The positioning of the flap is dependent on the patient’s dentition. If the third molar is present, its extraction facilitates the transition of the flap and prevents it from being damaged during chewing. In select cases, such as the reconstruction of the anterior portion of the tongue, the removal of the second molar may also be necessary. The Bozola technique involves a lower risk of the pedicle interfering with the occlusion at the level of the retromolar trigone and twisting and buckling when rotated 180° in the sagittal plane. In the rare case of occlusal interference, the risk of traumatism is easily resolved by using a temporary bite block. Following the adaptation of the flap to the intraoral defect, sutures are performed with absorbable material (vycril 3/0).

The donor area can be closed by either direct suture, in cases where the defect is small and favorably shaped, or by the buccal fat pad, in cases where the defect is greater than 3 cm (Figure 3D).

**Figure 4:**
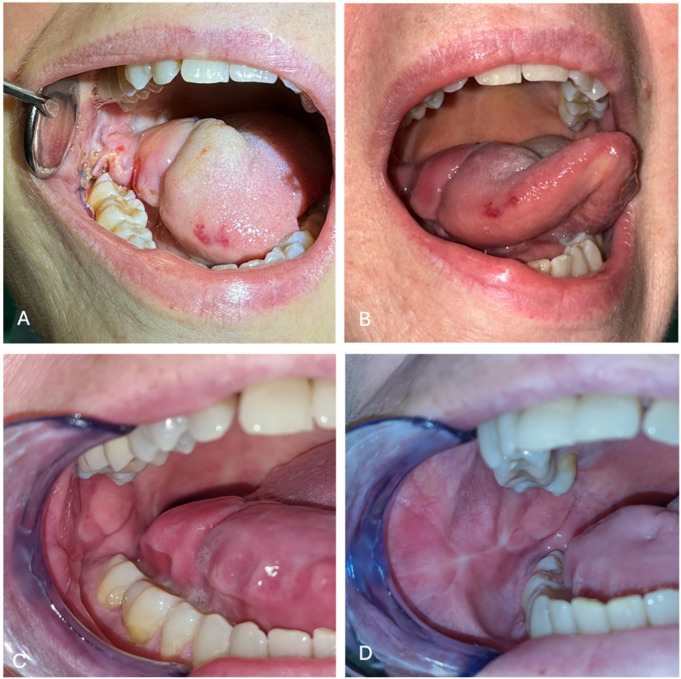
(**A**) Bozola flap one month after surgery. (**B**) Tongue motility, one week after the section of the vascular pedicle. (**C**) The acceptor site (oral tongue) three months after surgery. (**D**) donor site (cheek mucosa) three months after surgery.

The section of the pedicle under local anesthesia is performed three or four weeks after surgery, once neoangiogenesis has allowed the creation of a new vascular network on the flap. This procedure improves tongue mobility. Sometimes, this second step is not needed, and is provided only if there is no injury. Figure 4 highlights the patient’s postoperative clinical images. Figure 4A shows the patient one month after surgery, before the section of the pedicle, while Figure 4B presents lingual motility one week after the section of the pedicle. Figure 4C,D demonstrate the surgical site’s recovery after three months post-surgery.

### 2.4. Post-Operative Care

Patients are usually fed through a nasogastric tube for the first 48 h after surgery and then transitioned to a soft diet. The post-operative therapy includes standard short-course antibiotic prophylaxis (amoxicillin/clavulanic acid 875/125 mg for 72 h) and pain management [10]. On the fifth day after surgery, the patients are usually discharged. It is recommended that a soft food diet is followed for the following month. Routine follow-up based on head and neck tumor protocols should be performed.

Postoperative complications are rare but may include partial flap necrosis, which may require surgical revision; the dehiscence of the surgical site; and mild bleeding due to the detachment of the donor-suture site, which can be controlled by local packing and the possible use of anti-hemorrhagic drugs.

## 3. Discussion

The surgical excision of tumors affecting the oral cavity and the tongue invariably results in defects that significantly impact the morphology and functionality of the affected structures. In cases of extensive defects, free or locoregional flaps are typically employed for reconstruction, whereas smaller defects can be directly sutured [11]. Free flaps are indicated in patients with large defects, such as T3–T4 OTSCC patients, undergoing III, IV, and V types of glossectomy, according to Ansarin [3,4]. The employment of free flaps demands expertise in microsurgery, entails prolonged hospitalization, carries a risk of complications, and may result in adverse effects for the donor site. Although locoregional flaps provide a satisfactory amount of tissue for reconstruction, there are several drawbacks associated with their use. The flap tissue is primarily dissimilar to the resected flap, necessitating protracted operating times and an external incision that results in scarring on the donor site and the compromise of final aesthetic outcomes. Additionally, it can lead to trismus with constrained mouth opening. For intermediate-size defects, there are no clear standard indications. Myomucosal flaps are the appropriate option for repairing oral tongue cancer defects [12]. The Bozola flap has several advantages: it has characteristics similar to those of the removed tissues and maintains mucosal secretion and sensitivity, facilitating the rehabilitation of oral function in swallowing and speaking; it is elastic and malleable; it is reliable, thanks to the anatomical stability of its pedicle; it is easy and fast to perform; it has a wide arc of rotation that allows it to reach almost all of the oral cavity sites; there is a low morbidity of the donor site; it has no hair or skin appendages; and there is no primary or radiotherapy-associated shrinking of the flap.

A notable benefit of the Bozola flap in comparison to other myomucosis flaps relates to neck dissection. Indeed, some authors consider the presence of lymph node metastases to be a contraindication for the use of FAMM and Zhao flaps. The preservation of the facial artery could be a possible limitation of the cancer’s radicality in these patients.

Nonetheless, this technique is not without its limitations. Primarily, there is an insufficient amount of tissue available for large defects. Indeed, the Bozola flap does not apply to defects greater than 7 cm, and it is difficult to access the anterior parts of the oral cavity. Rare complications of this procedure include trismus, paralysis of the branch of the facial nerve, and incompetence of the labial commissure. Less rare are traumas to the pedicle, which, in some cases, necessitate the extraction of both the second and third molar to avoid trauma to the vascular peduncle. The extraction of these elements could cause chewing problems, periodontal problems, inflammation, and the reabsorption of the alveolar bone, as well as hepatology of the temporomandibular joint [12].

Lastly, the pedicle will be dissected during a second operation, which will be performed under local anesthesia. In some cases, a third surgery may be required to reduce the flap mass (interference with normal phonation and swallowing functions, or the presence of traumas and/or dental marks on the flap).

In our experience, the Bozola flap and other myomucosal flaps represent the ideal reconstructive option for the repair of oral cancer defects in patients undergoing type II and IIIa glossectomies following Ansarin classification. This myomucosal flap provides optimal results with respect to functionality, aesthetics, and patient comfort.

## 4. Conclusions

In conclusion, according to the literature, applying a buccinator myomucosal Bozola flap is a fast and reliable technique as an alternative to free flaps. Its wide range of rotation makes it suitable for managing defects of different shapes in the oral cavity. Based on our experience, the Bozola flap is recommended as the primary option for T1–T2 and certain T3 OTSCC defects (excluding the tip) for I–II and IIIa glossectomies in accordance with the Ansarin classification.

## Figures and Tables

**Table 1:** Classification of glossectomies proposed by Ansarin [9].

Type of Glossectomy		Anatomical Structures Included in Surgery
Type I	Mucosectomy	Mucosa and submucosa, including a thin layer of the intrinsic muscles.
Type II	Partial Glossectomy	Mucosa, submucosa, and intrinsic muscles up to the surface of the extrinsic muscles.
Type IIIa	Hemi-glossectomy	Surgery extends to all of the ipsilateral extrinsic muscles, including the lingual artery and the lingual and hypoglossal nerves.
Type IIIb	Compartmental Hemi-glossectomy	Surgery extends to all of the ipsilateral extrinsic muscles, including the genioglossus muscle, hyoglossus muscle, styloglossus muscle, and the lower portion of the palatoglossus muscle. The lingual nerve is resected as much as possible cranially, the hypoglossal nerve is removed after the ansa, and the lingual artery and vein are ligated in proximity to the hyoid bone.
Type IVa	Subtotal Glossectomy	This is an anterior-subtotal glossectomy with the preservation of both sides of the tongue base.
Type IVb	Near-Total Glossectomy	This is an anterior-subtotal glossectomy, including the ipsilateral base of the tongue; it preserves only the contralateral tongue base.
Type V	Total Glossectomy	It includes the mobile tongue, both sides of the tongue base, intrinsic and extrinsic muscles, both lingual arteries, hypoglossal, lingual nerves, and the floor of the mouth.

## Data Availability

The data is available on request from the corresponding author.

## References

[1] Molteni G., Ghirelli M., Molinari G., Presutti L. (2019). Microvascular reconstruction two years after subtotal glossectomy: Is it worth it?. J. Stomatol. Oral Maxillofac. Surg..

[2] Wang L., Liu K., Shao Z., Shang Z.J. (2016). Individual design of the anterolateral thigh flap for functional reconstruction after hemiglossectomy: Experience with 238 patients. Int. J. Oral Maxillofac. Surg..

[3] Canis M., Weiss B.G., Ihler F., Hummers-Pradier E., Matthias C., Wolff H.A. (2016). Quality of life in patients after resection of pT3 lateral tongue carcinoma: Microvascular reconstruction versus primary closure. Head Neck.

[4] Cortina L.E., Moverman D.J., Zhao Y., Goss D., Zenga J., Puram S.V., Varvares M.A. (2023). Functional considerations between flap and non-flap reconstruction in oral tongue cancer: A systematic review. Oral Oncol..

[5] Massarelli O., Baj A., Gobbi R., Soma D., Marelli S., De Riu G., Tullio A., Giannì A.B. (2013). Cheek mucosa: A versatile donor site of myomucosal flaps. Technical and functional considerations. Head Neck.

[6] Ferrari S., Balestreri A., Bianchi B., Multinu A., Ferri A., Sesenna E. (2008). Buccinator myomucosal island flap for reconstruction of the floor of the mouth. J. Oral Maxillofac. Surg..

[7] Bozola A.R., Gasques J.A., Carriquiry C.E., Cardoso de Oliveira M. (1989). The buccinator musculomucosal flap: Anatomic study and clinical application. Plast. Reconstr. Surg..

[8] Ferrari S., Copelli C., Bianchi B., Ferri A., Sesenna E. (2012). The Bozola flap in oral cavity reconstruction. Oral Oncol..

[9] Ansarin M., Bruschini R., Navach V., Giugliano G., Calabrese L., Chiesa F., Medina J.E., Kowalski L.P., Shah J.P. (2019). Classification of GLOSSECTOMIES: Proposal for tongue cancer resections. Head Neck.

[10] Iocca O., Copelli C., Ramieri G., Zocchi J., Savo M., Di Maio P. (2022). Antibiotic prophylaxis in head and neck cancer surgery: Systematic review and Bayesian network meta-analysis. Head Neck.

[11] Sittitrai P., Ruenmarkkaew D., Klibngern H. (2022). Pedicled Flaps versus Free Flaps for Oral Cavity Cancer Reconstruction: A Comparison of Complications, Hospital Costs, and Functional Outcomes. Int. Arch. Otorhinolaryngol..

[12] Remangeon F., Hivelin M., Maurice D., Lantieri L., Laccourreye O. (2017). The posterior-based buccinator myomucosal flap (Bozola’s flap). Eur. Ann. Otorhinolaryngol. Head Neck Dis..

